# Boosting Working Memory in ADHD: Adaptive Dual N-Back Training Enhances WAIS-IV Performance, but Yields Mixed Corsi Outcomes

**DOI:** 10.3390/brainsci15090998

**Published:** 2025-09-16

**Authors:** Alessandra Lintas, Michel Bader, Alessandro E. P. Villa

**Affiliations:** 1 NeuroHeuristic Research Group, University of Lausanne, Quartier UNIL-Chamberonne, 1015 Lausanne, Switzerland; 2 Laboratory for Behavioral Experiments (LABEX), University of Lausanne, Quartier UNIL-Chamberonne, 1015 Lausanne, Switzerland; 3 University Service of Child and Adolescent Psychiatry, University Hospital of Lausanne (CHUV), 1004 Lausanne, Switzerland; bader_m@bluewin.ch

**Keywords:** working memory training, dual N-back task, Attention-Deficit/Hyperactivity Disorder (ADHD), cognitive enhancement, executive function

## Abstract

**Background/Objectives:** This study investigates the efficacy of working memory training (WMT) using the dual N-back (DNB) task on cognitive performance in young adults with Attention-Deficit/Hyperactivity Disorder (ADHD). **Methods:** Over the course of at least 18 daily sessions conducted within one month, 106 participants (33 non-medicated ADHD, 42 medicated ADHD, and 45 controls) were randomly assigned to either a fixed dual 1-back (FD1B) training condition or an adaptive DNB condition, wherein the N-back level increased following successful completion of each trial block. Cognitive performance was assessed pre- and post-intervention using the Wechsler Adult Intelligence Scale–Fourth Edition (WAIS-IV) Working Memory Index (WMI) and the Corsi Block-Tapping Task. **Results:** A mixed-design ANOVA revealed significant improvements in DNB performance across all groups, with the adaptive training condition producing larger gains (e.g., a 204.6% improvement in controls, Cohen’s d=1.85). WAIS-IV WMI scores—particularly the Digit Span Backward subtest—also improved significantly post-training, with greater effect sizes in the adaptive condition (d=0.46) than in FD1B (d=0.27). Corsi performance showed very modest gains, showing a surprising tendency to be more associated with the FD1B condition than the adaptive condition. Control participants outperformed the medicated ADHD group on WAIS-IV subtests, although no significant differences emerged between medicated and non-medicated ADHD participants. Correlational analyses indicated task-specific training effects, with adaptive training enhancing associations between DNB and Corsi performance in both controls (r=0.60) and medicated ADHD participants (r=0.51). **Conclusions:** This study demonstrates that dual N-back training improves verbal working memory in young adults with ADHD, specifically in a sample without psychiatric comorbidities. Transfer benefit to visuospatial domains appears limited and may not generalize to adolescents, older adults, or individuals with complex clinical profiles. The results underscore the importance of tailoring training protocols to maximize cognitive outcomes across different domains.

## 1. Introduction

Attention-Deficit/Hyperactivity Disorder (ADHD) is a neurodevelopmental condition characterized by persistent inattention, hyperactivity, and impulsivity that impairs daily functioning [1]. It affects approximately 5–7% of children and persists into adulthood in about 60% of cases [2]. Cognitive theories, particularly Barkley’s executive function model, highlight working memory (WM) deficits as central to ADHD, affecting self-regulation and goal-directed behavior, and contributing to difficulties in academic and occupational domains [3]. WM encompasses both verbal and visuospatial components, which are often dissociated in ADHD; visuospatial impairments, in particular, are associated with difficulties in spatial organization and planning [4].

To clarify, WM requires storage and active manipulation of information (e.g., Digit Span Backward, Corsi Backward), whereas short-term memory (STM) refers to the capacity to hold information passively over a brief period (e.g., Digit Span Forward, Corsi Forward) [5,6]. Two widely used tasks for assessing the verbal and visuospatial subcomponents of WM are the Corsi Block-Tapping Task and the Working Memory Index (WMI) of the Wechsler Adult Intelligence Scale–Fourth Edition (WAIS-IV). The Corsi task evaluates visuospatial STM and WM by requiring participants to reproduce sequences of spatial locations in either the same (forward) or reverse (backward) order [6]. The forward condition primarily taps into STM, while the backward condition requires mental manipulation, thus indexing WM capacity. In individuals with ADHD, deficits are typically more pronounced in the backward span, indicating impaired visuospatial WM [4]. Furthermore, studies in older adults suggest that backward Corsi performance depends more on modality-specific subsystems than on the central executive, pointing toward selective visuospatial processing deficits rather than general executive dysfunction [7].

The WAIS-IV WMI targets verbal WM through subtests such as Digit Span, Arithmetic, and Letter-Number Sequencing [8]. In this context, Digit Span Forward primarily reflects short-term memory (STM), as it requires passive storage and repetition of sequences. In contrast, Digit Span Backward and Digit Span Sequencing demand active mental manipulation of numerical information, making them valid indicators of working memory (WM) capacity [5,8]. Similarly, Letter–Number Sequencing—which requires reordering alphanumeric sequences—places demands on both attention and WM processes. In adults with ADHD, WMI scores are typically lower than average, especially on subtests that involve complex WM operations and rapid processing, with moderate to large effect sizes reported in meta-analyses [9]. These flat cognitive profiles underscore relative WM weaknesses that persist despite average general intelligence [10]. However, the heterogeneity of ADHD presentations is also evident in studies showing that WMI performance has limited predictive power for symptom severity across individuals [11], reinforcing the importance of multi-dimensional assessments.

Despite the distinct modalities measured by Corsi and WAIS-IV, they are moderately correlated (r≈0.4), suggesting some overlap, but also conceptual divergence [6]. This dissociation supports theoretical models like the triple-pathway hypothesis, which posits that WM, inhibitory control, and temporal processing deficits can emerge independently across ADHD populations [12]. Thus, a multi-modal assessment approach is valuable for identifying individual cognitive profiles.

Interventions targeting WM deficits in ADHD have received significant attention. Among these, the dual N-back (DNB) task is a prominent training paradigm. Participants simultaneously monitor sequences of visual and auditory stimuli and respond to those presented n steps earlier, with difficulty adapting based on performance [13]. This task engages both verbal and visuospatial WM domains and activates prefrontal regions, supporting neural plasticity [14]. In healthy adults, DNB training has shown transfer effects, improving Digit Span performance on the WAIS-IV [15]. In children with ADHD, computerized WM training incorporating DNB paradigms led to 10–15% gains in visuospatial WM (Corsi backward) and reductions in ADHD symptoms [16]. Meta-analyses suggest that such cognitive training yields “near transfer” (regarding tasks closely related to training), but “far transfer” effects to general cognitive abilities or unrelated tasks (e.g., WAIS-IV subtests or Corsi task) remains inconsistent [17,18,19,20,21].

Indeed, some studies report only modest post-training gains in attentional control and minimal improvement on standard assessments like the WAIS-IV [22,23]. These mixed findings indicate that training effects are often task-specific and may not generalize beyond the trained modality [17,18,19]. Moreover, the sustainability of such improvements is questionable, particularly without continued training [17]. Nevertheless, the integration of both Corsi and WAIS-IV in assessments provides a more nuanced understanding of WM deficits and the specificity of training effects in ADHD.

To further explore these issues, we examined the effects of working memory training (WMT)—specifically DNB training—on performance in the WAIS-IV WMI and the Corsi task among young adults with ADHD and neurotypical controls. Individuals with ADHD were divided into two groups: a non-medicated ADHD group, including those who had never used pharmacological treatment or had stopped stimulant use after a suitable washout period; and a medicated ADHD group (MADHD), made up of participants on consistent stimulant medication for at least one month before and during the study. Notably, MADHD participants continued their medication until testing to maintain real-world treatment relevance. This study specifically focuses on young adults (aged 18–30) with ADHD and no major psychiatric comorbidities.

Participants were randomly assigned to either a baseline training condition (fixed dual 1-back; FD1B) or an adaptive training condition. The FD1B group completed daily sessions of the DNB task at a fixed difficulty level (1-back), ensuring consistent task exposure without WM load escalation. Conversely, the adaptive training group engaged in difficulty-adjusted sessions based on performance, increasing WM demands. This design controls for task familiarity and ensures that observed performance differences can be attributed to WM training rather than mere exposure. We hypothesized that only the adaptive group would show significant improvements in WM measures and that medication status would modulate the magnitude of these effects.

## 2. Methods

### 2.1. Participant Recruitment

We recruited 135 young adults (84 males and 51 females), aged between 18 and 30 years, who were distributed in three groups of 45 individuals each: individuals with ADHD treated with methylphenidate (MADHD), individuals with ADHD without medication (ADHD), and control (CTL). The ADHD group did not receive any pharmacological treatment during the study, having either never taken medication or had discontinued stimulant use for a sufficient washout period. In accordance with the study protocol, participants in the MADHD group (i.e., individuals with ADHD undergoing pharmacological treatment) were required to observe a 24 h washout period prior to laboratory assessments. This practice is commonly adopted in cognitive neuroscience studies to reduce the influence of acute stimulant effects and better isolate trait-level cognitive performance. Importantly, this approach was mandated by the Ethics Committee, which stipulates that deviations from standard clinical procedures must be medically justified. Although this strategy aims to ensure standardized testing conditions across participants, we recognize that stimulant discontinuation may lead to transient symptom rebound or reduced attentional engagement during testing. Control participants were age-/education-matched volunteer healthy controls screened prior to the experimental session to ensure that they would not report any neuropsychiatric diseases or any other exclusion criteria, and none were taking any psychoactive medications.

Under the supervision of a trained clinical psychologist, all participants underwent the Mini-International Neuropsychiatric Interview [24], a short structured diagnostic interview assessing psychiatric diseases, in order to exclude from this study those with ADHD comorbidities. The exclusion criteria also included any individual with a history of sustained head injury and any individual referred taking mood and anxiety stabilizers, anti-depressants, any dopamine receptor-blocking drug, or non-stimulant medications for ADHD. All patients were recruited among patients diagnosed with ADHD [25] clinically referred either by the Psychiatric Department of the University Hospital of Lausanne or at a psychiatrist’s practice in collaboration with the University Hospital. This study was carried out in accordance with the latest version of the Declaration of Helsinki [26], and was approved by the mandatory Ethics Committees requested by Swiss Federal Authorities, following the constitutional article (art. 118b Cst) of 8 March 2010 and the Federal Act involving Human Beings on 30 September 2011 (revised 1 January 2014). All participants had normal or corrected-to-normal visions and received monetary compensation following the scale approved by the mandatory Ethics Committees requested by Swiss Federal Authorities. They were requested to fill out French versions of the adult ADHD Self-Report Scale (ASRS) [27] and the Conners’ Adult ADHD Rating Scales-Self Report (Screening Version, CAARS-S:SV) [28] two weeks prior to the beginning of the protocol.

The participants of all groups were further divided into subgroups based on the type of at-home training condition, being randomly assigned to a fixed-D1B or adaptive DNB training. The assignment of a participant to the ‘medicated ADHD group’ (MADHD) or to the ‘ADHD group without medication’ (ADHD) was decided by the psychiatrist in charge of the patient on the exclusive basis of the patients’ therapeutic treatment. This assignment was ‘double-blind’, in the sense that the experimenters did not know which patient belonged to either ADHD group until the end of the protocol and the psychiatrist did not know which level of working memory training was assigned to a patient.

### 2.2. Dual N-Back Task (DNB)

The dual N-back task used in this study was a French adaptation of the paradigm originally proposed by [13,15]. The task was programmed to include 20 blocks of 20 + N trials, with N indicating the current difficulty level. Participants initiated each block manually by pressing a key. Each trial consisted of a simultaneous presentation of visual and auditory stimuli, requiring participants to divide their attention across modalities and continuously update and compare each incoming stimulus to the ones presented earlier in N trials.

Participants maintained gaze on a central fixation cross displayed on a 19-inch monitor (viewing distance ∼70 cm). Visual stimuli were blue squares (3.8 × 3.8 cm) randomly appearing in one of eight spatial locations arrayed in a 3 × 3 grid (excluding the center). Participants were instructed to press the “A” key whenever a square appeared in the same location as the one in the earlier N trials. Auditory stimuli consisted of letters (Q, D, H, G, K, M, R, Z) spoken by a female voice. A match in the auditory stream (i.e., the same letter as in the N trials) required a response via the “L” key.

**Figure 1:**
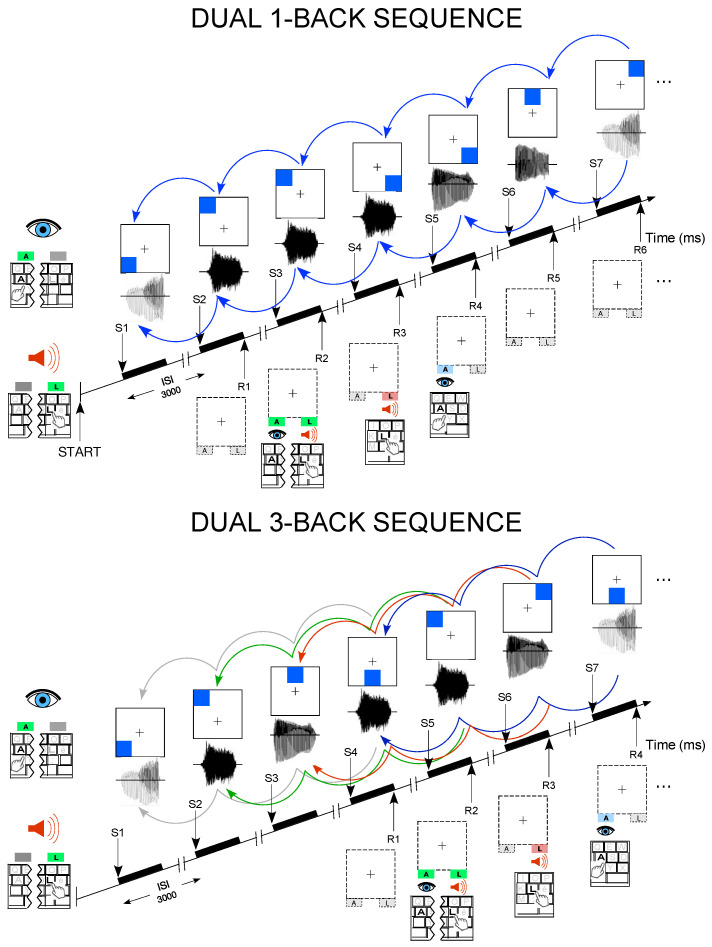
Experimental design of the dual 1-back and dual 3-back. At each trial, the participants were asked to press the “A” key for the visual modality and/or the “L” key for the auditory modality if the stimulus in either modality is identical to the cue presented 1-trial back (**upper panel**) and 3-trials back (**bottom panel**) in time. This example illustrates the four response conditions occurring in the task: R1: incongruent stimuli in either modality—no key press (dual incongruent); R2: auditory and visually congruent stimuli; R3: auditory congruent and visually incongruent stimuli; R4: auditory incongruent and visually congruent stimuli.

The task comprised four response conditions per trial (Figure 1); R1: no match in either modality—no key press (dual incongruent); R2: match in both modalities—press both “A” and “L” simultaneously (dual congruent); R3: auditory-only match—press “L” (auditory congruent, visual incongruent); R4: visual-only match—press “A” (visual congruent, auditory incongruent). This structure ensured constant engagement of both verbal and visuospatial working memory (WM) systems across all levels of difficulty. The same N value applied to both auditory and visual modalities within a given block.

At the start of training, participants performed a dual 1-back block to familiarize themselves with the task and response contingencies. Immediate feedback was given after each trial via color-coded feedback (green for correct, red for incorrect). Each trial lasted 3000 ms. Visual and auditory stimuli were presented concurrently for 500 ms, and participants had up to 2500 ms to respond. Missed responses were counted as errors. The session lasted approximately 25–35 min, depending on block difficulty and break duration.

Task difficulty was regulated using an *adaptive staircase algorithm* modeled on validated procedures from [13,14,15], wherein the N-back level increased or decreased based on performance in the preceding block: (1) if a participant made fewer than 3 errors in total (combined across both modalities), the difficulty increased by 1 (N → N + 1); (2) if a participant made 5 or more errors in either modality, the difficulty decreased by 1 (N → N − 1); and (3) if performance was intermediate (3–4 errors total, but fewer than 5 in either modality), the N level remained unchanged. This rule ensured a challenge tailored to individual capacity, minimizing ceiling or floor effects. The algorithm allowed for N values to vary dynamically between blocks, typically ranging from 1 to 6. Each block comprised 20 + N trials to allow for sufficient adaptation time.

### 2.3. Working Memory Training Protocol

In a pre- and post-intervention session, at the laboratory, all participants played the adaptive version of the DNB task. The WM training started the day after the pre-training session. At home, the participants played DNB by mean of an Internet remote connection to a server with protected access. The training structure, based on 20-block daily adaptive dual N-back sessions, aligns with protocols previously validated for adult ADHD populations [21]. The strict requirement was to complete at least 18 training sessions over four weeks. Randomly assigned participants in both the control and ADHD group were requested to perform a WM training either with a fixed 1-back level of difficulty (FD1B) or with a performance-dependent adaptive N-level of difficulty. In both sessions, the participants performed the WAIS-IV (Wechsler Adult Intelligence Scale—Fourth Edition) digit span subtest from the Wechsler Adult Intelligence Scale, which requires participants to sequentially order the numbers (i.e., backward, forward, and sequencing digit span) presented by the examiner [8], and the forward span of the Corsi block-tapping task, which is a visuospatial short-term memory task [7].

### 2.4. Statistical Analysis

The R framework version 4.4.3 [29,30] was used for all statistical analyses, using packages including rstatix, EnvStats, lme4, lmerTest, afex, effectsize, emmeans, tidyverse, psych, and Superpower for power simulations. To ensure the robustness of the statistical conclusions, an outlier analysis was conducted across all groups and cognitive variables using Rosner’s test. The Shapiro–Wilk test was used to test deviations from normality with a level of significance of p=0.05. Descriptive statistics are reported as the mean ± standard error of the mean (SEM), unless otherwise specified. Independent tests are used to assess pre-intervention baseline differences (age, handedness, and gender) between the groups. We ran a one-sample *t*-tests to compare each group’s mean improvement rate (%Δ) against a reference value of 100% (indicating no change).

A linear mixed-effects model (LMM) was used to examine changes in DNB level score over training days across three groups (CTL, ADHD, and MADHD). The model included the fixed effects for the group, day (treated as categorical), and their interaction, with a random intercept and slope for each participant. A mixed-design three-way ANOVA was conducted for each behavioral measurement with one within-subject factor “session” (pre-/post-intervention with WMT) and two between-subject factors: “Group” (CTL/ADHD/MADHD) and “Training” condition (adaptive/fixed-D1B). Effect size is computed by $\eta_{G}^{2}$, which is more conservative and comparable across different designs. Post hoc *t*-tests were computed to identify the factor that was significantly different between the groups. In addition, raw comparisons from pre- to post-intervention were analyzed using paired-samples *t*-tests within each group and training condition. Cohen’s *d* was computed to assess the effect size for all *t*-tests. Finally, we ran Pearson correlation analyses to investigate the association between cognitive domains across the participants’ groups.

## 3. Results

### 3.1. Sample Demographics Analyses

Of the 135 participants initially recruited, 15 did not complete the at-home working memory training (WMT) protocol. These dropouts included twelve unmedicated ADHD participants (six in fixed-D1B and six in adaptive training) and three medicated ADHD participants (two in fixed-D1B and one in adaptive training).

Table 1 presents the demographic and clinical characteristics (age, gender, handedness, ASRS score, and ADHD Index) of the remaining 120 participants across groups. There were no significant group differences in age, gender, or handedness (Age: F(2,117)=0.47, p=0.63; Gender: $\chi^{2}\left(2,N=120\right)=3.52$, p=0.17; Handedness (EHI): F(2,117)=1.64, p=0.20). However, comparison between the two clinical groups (ADHD and MADHD) revealed significant differences in self-reported symptom severity: ASRS total scores differed significantly (F(1,73)=8.37, p=0.005), as did the CAARS ADHD Index scores (F(1,73)=6.10, p=0.016, $\eta_{G}^{2}=0.087$). Participants in the MADHD group scored higher on both measures, suggesting greater perceived symptom burden despite ongoing pharmacological treatment.

The difference in ADHD Index scores reflects a moderate symptom burden in both groups, with the medicated group reporting unexpectedly higher levels. Table 2 displays the distribution of ADHD diagnostic subtypes. No significant group differences were observed in subtype frequency, $\chi^{2}\;\left(3,N=75\right)=4.81$, p=0.19. These results indicate that differences between the MADHD and ADHD groups are likely to reflect the effects associated with the pharmacological treatment administered to the MADHD participants.

We excluded an additional 14 participants from the main inferential analyses due to extreme pre-to-post training performance changes, identified using Rosner’s generalized extreme Studentized deviate (ESD) test for multiple outliers. These cases were not related to missing data, but rather reflected statistically atypical patterns based on robust deviation thresholds. The rationale for exclusion is that the pre- and post-training DNB assessments were conducted in the laboratory, where some participants may have been distracted or insufficiently engaged, leading to implausible performance shifts. The excluded cases comprised six control participants: two from the ADHD group and six from the MADHD group. This conservative approach was implemented to reduce distortion in group-level analyses caused by participants with aberrant trajectories, which may result from reduced task engagement, technical artifacts, or protocol non-compliance. Notably, exclusions were distributed evenly across groups to minimize potential bias. The final sample for analysis included 106 participants: 39 controls, 31 ADHD, and 36 MADHD.

### 3.2. Working Memory Performance Across Training Days

Before training, a two-way ANOVA was conducted to examine the effects of group (CTL, ADHD, MADHD) and training assignment condition (fixed-D1B vs. adaptive) on pre-training DNB level scores in order to assess potential baseline differences. There were no significant main effects of group (F(2,100)=1.94, p=0.15) or training assignment condition (F(1,100)=1.86, p=0.18). Additionally, the group × training interaction was not significant (F(2,100)=0.98, p=0.38), and Levene’s test confirmed the homogeneity of variances (F(5,100)=0.75, p=0.59). These results confirm the absence of significant baseline differences in DNB scores between groups and training conditions. While non-significant, this outcome is desirable, as it suggests statistical equivalence at baseline, permitting clearer interpretation of post-training effects without confounding from pre-training disparities.

**Figure 2:**
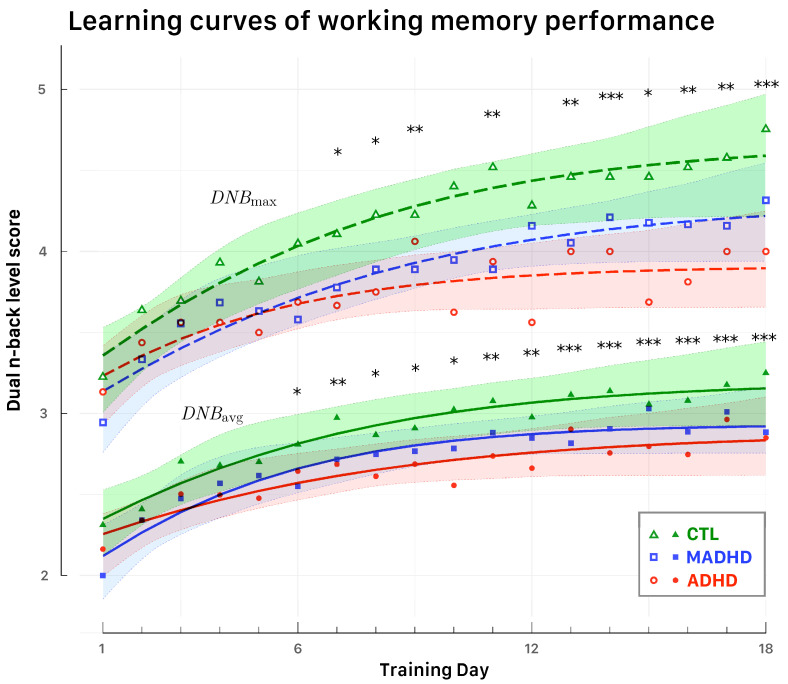
Learning curves of working memory performance across training days. The figure displays logistic fits for average (*DNB*_avg_) and peak (*DNB*_max_) performance across 18 daily training sessions. Group-wise means are overlaid for CTL (triangles), ADHD (circles), and MADHD (squares). Shaded bands denote 95% confidence intervals. Statistically significant improvements over baseline (from LMM analyses) are marked along the days with asterisks (* p<0.05; ** p<0.01; *** p<0.001).

For each participant, we recorded DNB level scores during each block of the dual N-back (DNB) adaptive training conducted at home over 18 daily sessions. Figure 2 displays changes in average (*DNB*_avg_) and peak (*DNB*_max_) performance over time.

To evaluate training efficacy, a first analysis compared scores at two time points: the beginning (Day 1) and end (Day 18) of training. A mixed-design two-way ANOVA with within-subject factor “Day” (Day 1 vs. Day 18) and between-subject factor “Group” (CTL, ADHD, MADHD) revealed the significant main effect of Day (minimum F(1,47)=33.13, p<0.001, $\eta_{G}^{2}=0.19$), indicating a robust improvement from pre- to post-training. Neither the main effect of Group (F(2,47)=0.87, p=0.42) nor the Day × Group interaction (F(2,47)=0.80, p=0.45) were significant.

To characterize the trajectories of change across all training days, a linear mixed-effects model (LMM) was applied to both *DNB*_avg_ and *DNB*_max_ scores, with fixed effects for Group, Day, and their interaction, and random slopes and intercepts for participants. Significant gains were observed over time for both metrics: *DNB*_avg_: F(17,646)=13.94, p<0.001, $\eta_{G}^{2}=0.06$; and *DNB*_max_: F(17,646)=9.88, p<0.001, $\eta_{G}^{2}=0.06$. No significant main effects of Group or Group × Day interactions were observed, suggesting that learning rates were statistically similar across diagnostic categories.

### 3.3. DNB Level Scores After Working Memory Training

**Figure 3:**
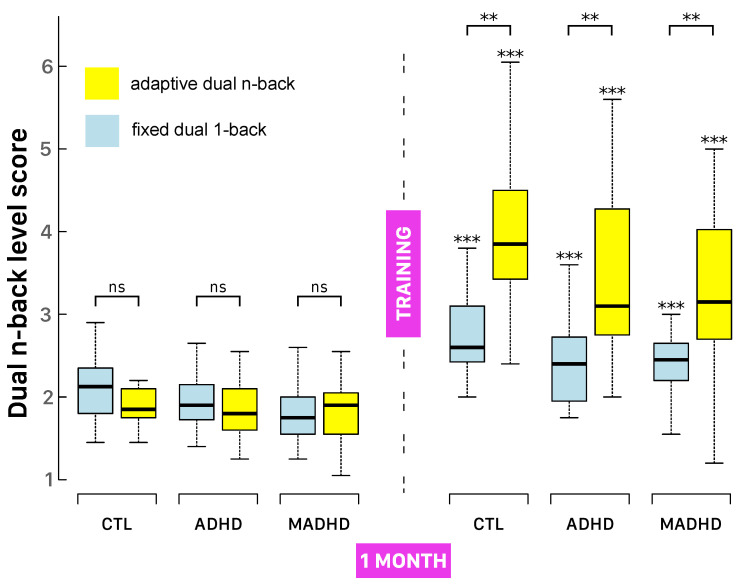
Comparison of pre- and post-intervention performance as a function of the training condition. Box-and-whisker plots display DNB level scores before and after training across both fixed-D1B (light blue) and adaptive (yellow) training protocols. For each group (CTL, ADHD, MADHD), the boxes span the interquartile range (IQR), the thick horizontal line marks the median, and whiskers extend to 1.5 × IQR. For each group and for each training protocol, statistically significant improvements over baseline (from pairwise two-sample *t*-test) are marked above the whiskers with asterisks (***: p<0.001). Bracket markers are used for each group’s comparison (Welch’s *t*-test) between the adpative and fixed dual 1-back training samples, with statistical significance (ns: p>0.05; **: p<0.01).

All groups demonstrated statistically significant improvements in DNB level scores from pre- to post-intervention across both training protocols. As shown in Figure 3 and Table 3, participants in both the ADHD and MADHD groups reached at least level 2 (i.e., successfully completing a 2-back task without errors) by the end of training.

A three-way mixed-design ANOVA confirmed the robust main effect of Session (F(1,100)=283.55, p<0.001, $\eta_{G}^{2}=0.39$), indicating substantial overall gains in DNB performance. Adaptive training led to greater improvements than fixed-D1B (F(1,100)=11.55, p<0.001, $\eta_{G}^{2}=0.08$), with a highly significant Session × Training interaction (F(1,100)=65.32, p<0.001, $\eta_{G}^{2}=0.13$), demonstrating that adaptive training produced larger performance increases. Group-wise improvements in DNB level scores under adaptive training were as follows: CTL 2.01±0.22, ADHD 1.58±0.30, MADHD 1.50±0.22; and under fixed-D1B: CTL 0.75±0.14, ADHD 0.46±0.12, MADHD 0.58±0.09. All gains were statistically significant (see asterisks in Figure 3).

Percentage improvement (Table 3) and effect sizes were also robust: controls under adaptive training improved by 204.6%±13.0 (t(18)=8.05, p<0.001, d=1.85), compared to 136.4%±4.3 under FD1B (t(19)=8.52, p<0.001, d=1.91). Similar patterns were seen in ADHD and MADHD groups (all p<0.001, d>1.6), confirming strong training effects. While all groups benefited (F(2,100)=3.50, p=0.034, $\eta_{G}^{2}=0.04$), post hoc tests showed that control participants outperformed MADHD, suggesting differential responsiveness to training and possible residual or treatment-resistant deficits in MADHD.

### 3.4. WAIS-IV Working Memory Index

The WAIS-IV working memory improvement analysis focused on subtests directly aligned with the standard WAIS-IV Working Memory Index, as shown in Table 3. Specifically, we examined Digit Span Forward, Digit Span Backward, Digit Span Sequencing, and the composite Total Working Memory Score. A three-way mixed-design ANOVA revealed significant main effects of Session and Group across multiple WAIS-IV measures. The smallest observed Session effect was for Digit Span Sequencing (F(1,100)=7.54, p=0.007, $\eta_{G}^{2}=0.02$), while the smallest significant Group effect occurred for Digit Span Forward (F(2,100)=3.97, p=0.022, $\eta_{G}^{2}=0.06$). These results suggest overall post-training improvements in working memory, as well as baseline group differences, particularly for Digit Span Forward.

Post hoc analyses revealed that adaptive training yielded significantly greater gains than fixed-D1B training on key WAIS-IV metrics. For Digit Span Backward, adaptive training led to a larger improvement (t(100)=4.77, p<0.001, d=0.47) compared to the fixed-D1B condition (t(100)=2.84, p=0.006, d=0.28). Similarly, the Total Working Memory Score improved substantially under adaptive training (t(100)=4.61, p<0.001, d=0.46), while fixed-D1B showed more modest gains (t(100)=2.76, p=0.007, d=0.27). Group comparisons revealed that control participants significantly outperformed the MADHD group on all WAIS-IV subtests (all p<0.05), while no significant differences emerged between the ADHD and MADHD groups (all p>0.40).

Our findings suggest that while adaptive training is particularly effective in enhancing working memory performance, ADHD-related deficits—especially among medicated participants—persist even after the intervention. The participants completed the adaptive dual N-back (DNB) task during both pre- and post-intervention laboratory sessions, regardless of their assigned training condition. Although this approach ensured consistent task exposure across individuals, it may have introduced a short-term activation effect that influenced WAIS-IV performance. Consequently, some of the observed improvements in verbal working memory—particularly in Digit Span subtests—may reflect acute priming from immediate engagement with the adaptive DNB task, in addition to genuine long-term training effects.

### 3.5. Corsi Block-Tapping Task

Visuospatial memory was assessed by comparing pre- and post-intervention performance on the Corsi Block-Tapping Task (Table 3), focusing on two key measures: the Corsi Span Length, which reflects visuospatial short-term memory capacity, and the Corsi Number Correct, indicating the total number of accurately completed trials. A significant main effect of Group was found only for Corsi Span Length (F(2,100)=3.44, p=0.036, $\eta_{G}^{2}=0.05$). On the contrary, a main effect of Session was significant for Corsi Span Length (F(1,100)=8.64, p=0.004, $\eta_{G}^{2}=0.02$) and for Corsi Number Correct (F(1,100)=13.99, p<0.001, $\eta_{G}^{2}=0.02$), indicating overall improvement following the intervention. However, no consistent session-by-group or session-by-training interactions were observed, suggesting that these improvements were not specific to particular groups or training conditions.

For the Corsi Number Correct, irrespective of the group, we observed a significant improvement rate following fixed-D1B training (t(51)=3.62, p=0.002, d=0.50), whereas improvement under the adaptive training protocol was marginal (t(53)=1.75, p=0.037, d=0.31). A similar trend was observed for the Corsi Span Length: fixed-D1B training produced a moderate and significant improvement rate (t(51)=3.31, p=0.004, d=0.46), while adaptive training yielded a non-significant gain (t(53)=1.48, p=0.15, d=0.20).

### 3.6. Correlations Among Cognitive Domains

To explore the dynamics of cognitive interrelations, we computed Pearson correlation coefficients for each combination of training condition, diagnostic group, and pre-/post-testing phase. All participant groups showed internal consistency in WAIS-IV measurements before and after WMT, irrespective of the training condition, particularly with a strong stable correlation between WAIS-IV Digit Span Backward subtest and WAIS-IV Total Working Memory Score (r>0.97, p<0.001). Other subgroup-level correlations revealed nuanced patterns of associations that differ depending on context. Specifically after adaptive training, controls were characterized by stronger positive correlations among cognitive variables, particularly DNB level linked with Corsi (r=0.60, p=0.007) and, to a lesser extent, WAIS-IV sequencing with Corsi (r=0.54, p=0.017). In ADHD participants, a moderate correlation between DNB level and the other cognitive domains observed before WMT tended to weaken after either fixed-D1B or adaptive training. On the contrary, the MADHD group showed that DNB level was correlated with the Corsi task (r=0.51, p=0.001), as well as with WAIS-IV measurements (r=0.49, p=0.003), before intervention. In this group, the best correlation between improvement rates (%Δ) of DNB and Corsi performances was associated with FD1B (r=0.58, p=0.014). Collectively, these results suggest that cognitive improvements in participant groups tended to occur independently across domains, and training type (adaptive vs. FD1B) had no significant impact.

## 4. Discussion

This study examined the efficacy of working memory training (WMT) using the dual N-back task in young adults with ADHD, comparing adaptive and fixed-D1B training conditions on WAIS-IV Working Memory Index (WMI) and Corsi Block-Tapping Task performance. Our results indicate that working memory scores improved after training, particularly under adaptive conditions and variably across groups. All groups displayed improvements in working memory performance, particularly measured by N-level gains in the in dual N-back task. Gains in traditional cognitive measures (WAIS-IV, Corsi) were present but less uniform, and should be interpreted with more caution. The results with controls suggest the tendency to transfer effects to broader working memory domains. Observed visuospatial working memory benefits in ADHD patients may depend on specific group–training interactions, with potential advantages for medicated clinical populations. Despite the observed gains in working memory performance, it is important to acknowledge a key limitation of the present design: the absence of a passive, no-intervention control group.

It is important to note that our participants’ clinically diagnosed patients samples show that medicated individuals (MADHD) reported higher ASRS total scores despite medication, as well as a differences in ADHD Index scores from the CAARS. Both scores were, on average, below the clinical cut-off associated with clinically elevated ADHD symptoms, thus reflecting a moderate symptom burden in both ADHD and MADHD groups, with the medicated group reporting higher levels than expected. These findings are somewhat counterintuitive, as medicated individuals reported equal or greater symptom severity compared to the non-medicated ADHD group.

An important interpretive consideration arises from the 24 h stimulant washout enforced for the MADHD group. This washout period is meant to be safe, minimally disruptive, and sufficient to eliminate the immediate pharmacological effects of most stimulants, as their half-life ranges from 2–7 h for methylphenidate and 10–13 h for amphetamines [31]. Although standard in research protocols aiming to avoid acute pharmacological effects during testing [32,33], this requirement may have introduced unintended confounds, even if it facilitates comparisons with the prior literature and meta-analyses. Elevated ASRS scores in this group at baseline could reflect temporary withdrawal effects or unmedicated symptom expression, rather than stable trait differences. Additionally, reduced attention capacity during testing could have influenced task engagement and performance metrics. Future studies should consider alternative approaches, such as within-subject crossover designs or longer washout intervals, to better isolate medication effects while minimizing transient rebound phenomena [34,35,36,37]. In this study, ADHD participants did not receive any pharmacological treatment, having either never taken medication or had discontinued stimulant use for a sufficient washout period. On the opposite, individuals receiving pharmacological treatment for ADHD (i.e., MADHD) may report more severe symptoms on self-report measures, potentially reflecting more persistent symptomatology, increased self-monitoring and insight resulting from treatment engagement, or symptoms rebound due to sudden discontinuation.

A three-way mixed design ANOVA revealed significant main effects from group, training protocol, and session on DNB level scores, as well as a significant training-by-session interaction, supporting the effectiveness of adaptive training (e.g., 204.6% improvement in controls, Cohen’s d=1.85). General eta-squared estimates indicated medium to large effect sizes ($\eta_{G}^{2}=0.13$ for the Training × Session interaction and $\eta_{G}^{2}=0.39$ for the main effect of Session). WAIS-IV WMI scores, particularly Digit Span Backward, showed robust post-training enhancements, with adaptive training producing greater effect sizes (d=0.46) than FD1B (d=0.27). Controls outperformed medicated ADHD participants on WAIS-IV subtests, with no significant differences between ADHD groups. These results align with prior research indicating that WMT can enhance working memory (WM) capacity, particularly in verbal domains, but highlight limited transfer to visuospatial tasks in ADHD populations [15,38]. During a gambling task, ERP measures in ADHD identified altered frontocentral activity in reward-related tasks, which may intersect with attention modulation pathways engaged during working memory training [39]. Our findings support earlier reports that adults with ADHD undergoing four weeks of adaptive dual N-back training showed significant gains in the conflict effect of the Attention Network Task (ANT) [21]. Notably, these gains were correlated with lower baseline hyperactivity-impulsivity scores and were independent of psychostimulant medication use. This interpretation aligns also with ERP findings showing enhanced early attentional modulation (indexed by P1 waveforms) in ADHD patients during a gambling task, but only under high WM load conditions [40]. This suggests that training-induced improvements in executive control may be modulated more by individual trait profiles than by pharmacological factors, highlighting the importance of personalized cognitive interventions in ADHD management [21].

### 4.1. Improvements in Dual N-Back Performance

The substantial gains in DNB performance, especially under adaptive conditions, support the task’s efficacy in engaging both verbal and visuospatial WM systems [13]. Adaptive training’s superiority reflects its ability to dynamically adjust difficulty, maintaining optimal cognitive load and fostering neural plasticity in prefrontal and parietal regions [14]. The large effect sizes (e.g., *d* from 1.66 to 3.02) corroborate findings from Jaeggi et al. [15], who reported transfer effects to untrained WM tasks in healthy adults. In ADHD, similar improvements in children [16], although our study extends these findings to adults, suggest that adaptive WMT remains effective across developmental stages. However, the lack of significant group differences between non-medicated (ADHD) and medicated (MADHD) participants indicates that stimulant medication may not substantially modulate WMT outcomes, aligning with Cortese et al. [17], who found a limited interaction between medication and cognitive training efficacy.

### 4.2. WAIS-IV WMI Outcomes

The improvements in WAIS-IV WMI, particularly Digit Span Backward, underscore the sensitivity of verbal WM to WMT. Adaptive training’s larger effect sizes suggest that increasing task demands enhances WM manipulation skills, critical for ADHD individuals who exhibit deficits in self-regulation [3]. However, we now acknowledge a potential confound in our design: all participants, regardless of their home training condition, completed the adaptive dual N-back task during both pre- and post-intervention laboratory sessions. While this ensured a consistent working memory performance probe, it also introduced the possibility of short-term task priming or activation effects influencing WAIS-IV results. Therefore, the observed improvements in verbal working memory—particularly in Digit Span subtests—may reflect a combination of sustained training gains and immediate engagement with the adaptive task. Similar short-term transfer effects have been reported in the literature [40,41]. Caution is therefore warranted in attributing WAIS-IV improvements solely to the assigned training protocol.

The control group’s superior performance over MADHD participants supports a “spiky” cognitive profile in ADHD, where relative WM weaknesses persist despite medication [10]. Notably, the absence of differences between ADHD and MADHD groups contrasts with previous findings showing the limited predictive power of WAIS-III WMI for ADHD symptom severity [11]. This discrepancy may reflect WAIS-IV’s enhanced sensitivity to WM manipulation in our ADHD diagnosed groups via subtests like Digit Span Sequencing [42].

### 4.3. Corsi Task Findings

Despite expectations that adaptive training would yield greater improvements, the counterintuitive finding that fixed-D1B training tended to outperform adaptive training on Corsi performance could suggest that visuospatial working memory benefits may depend on specific group–training interactions. A modest Group effect was found only for Corsi Span Length and the Session × Training interaction was not statistically significant for Corsi outcomes, and thus, gains in the Corsi Block-Tapping Task cannot be reliably attributed to the training protocol per se. Dual 1-back training condition might have allowed participants to develop task-specific strategies, inflating Corsi Span Length and Number Correct scores without taxing WM capacity [6]. This aligns with Melby-Lervåg et al. [23], Shipstead et al. [43], who noted that WMT effects are often task-specific, with limited transfer to untrained visuospatial tasks in ADHD. However, we acknowledge that the emergence of procedural strategies or practice-related improvements in visuospatial WM [44] remain speculative for our results. Additionally, we did not collect qualitative or process-level data (e.g., strategy use, motivation, or reaction times) that could support interpretations involving cognitive strategies or engagement patterns. As such, we caution against causal interpretations, and instead frame these findings as unexpected, warranting further investigation in future studies designed to evaluate underlying mechanisms more directly.

### 4.4. Limitations of This Study

The strength of this study is the assessment of a “baseline” effect of working memory training produced by fixed-D1B. Nonetheless, the limitations of this design should also be considered, particularly the lack of an additional no-training control group, thus precluding the definitive attribution of improvements to WMT versus time or test familiarity. While the fixed-D1B condition was intended to serve as a structurally comparable low-intensity intervention control, it cannot fully rule out potential test–retest or expectancy-related improvements. This is particularly relevant for tasks like the Corsi Block-Tapping test, where procedural familiarity and practice effects might partially explain post-training gains [43]. In addition, the study’s focus on young adults limits generalizability to children or older adults with ADHD, where WM profiles may differ [45]. Thus, although we might interpret the observed improvements as evidence of a training effect, caution is warranted in attributing these entirely to the intervention in the absence of a non-active comparator condition.

Working memory interventions are known to interact with comorbidity profiles [46,47]. Therefore, our findings should be interpreted within the context of our specific sample, which consisted exclusively of young adults aged 18–30 years with clinically diagnosed ADHD but without major psychiatric comorbidities or neurodevelopmental disorders beyond ADHD. We acknowledge that conclusions are limited to this age group and clinical profile. As ADHD symptomatology and working memory capacity evolve developmentally, the applicability of these results to adolescents or older adults remains untested. Moreover, the exclusion of individuals with common comorbidities, such as anxiety or learning disorders, reduces broader generalizations. Future studies should examine the generalizability of adaptive training protocols across a more diverse clinical spectrum.

To assess the sensitivity of the current design, a post hoc simulation-based power analysis was conducted using the Superpower package in R [48], which allows for complex factorial designs involving repeated measures. While traditional tools such as G*Power [49] (version 3.1.9.8) are limited to simpler models, simulation-based estimates (1000 iterations, α=0.05) offer greater flexibility in modeling complex repeated-measures designs and empirical variance structures. Such a power analysis tuned to the smallest group sample size (n=15) revealed strong power to detect the main effect of Session (1−β=1.00) with a very large effect size (Cohen’s f=0.76) on DNBlevel, indicating a strong likelihood of detecting overall change from pre- to post-assessment. The interaction between the training protocols and Session was also well-powered (1−β=1.00, f=0.43), supporting sensitivity to differences in pre–post change as a function of training condition. WAIS-IV working memory index measurements demonstrated adequate power to detect within-subject changes over time (Session effect) and group differences (in particular WAIS-IV Total Working Memory Score, with power 1−β=0.96, f=0.15, and power 1−β=0.84, f=0.13, respectively). However, the power to detect the main effect of session (1−β=0.79, f=0.094) was just acceptable on measurements of Corsi Block-Tapping Task.

We acknowledge that the limited sample size may have reduced power to detect subtle group differences, particularly in the MADHD group, where medication dosage variability could influence outcomes [50]. Moreover, the 4-week duration of the intervention, though consistent with prior studies [15,17], may be insufficient to produce lasting or generalized effects across cognitive domains. Several meta-analyses and critical reviews have highlighted that short-term WM training often produces short-lived or task-specific improvements, with limited evidence supporting sustained transfer beyond the trained tasks or domains [23,43,51]. While the observed post-training gains suggest the effectiveness of adaptive training in modulating WM processes in the short term, future studies should investigate whether these effects persist over longer time frames and whether extended training durations (e.g., 8–12 weeks) yield stronger or more durable transfer. The absence of long-term follow-up in our design further limits conclusions regarding retention or delayed transfer effects.

### 4.5. Clinical Implications and Future Directions

Our findings have potential implications for cognitive interventions targeting working memory in young adults with ADHD without psychiatric comorbidities. Caution is warranted in generalizing these results to broader ADHD populations, such as adolescents, older adults, or those with comorbid mood, anxiety, or learning disorders. Prior work has shown that training outcomes can interact with individual clinical profiles and developmental stage [46,47].

Adaptive dual N-back training’s efficacy in enhancing verbal WM suggests its potential as a non-pharmacological adjunct to improve academic and occupational outcomes [17]. The training-related improvements are probably not due solely to a placebo effect and are probably not simply driven by positive expectancies [16,52,53,54]. However, the limited transfer to visuospatial WM, critical for spatial organization in ADHD [4], underscores the need for multimodal training protocols. Combining dual N-back with visuospatial-specific tasks, such as virtual reality-based exercises [55,56], and metacognitive intervention to supplement working memory training [57], could address this gap. These new interventions should also target additional processes and be embedded within more focused intervention strategies [54]. The lack of medication-related differences suggests that WMT benefits are accessible to both medicated and non-medicated individuals, enhancing its ecological validity. Individual variability in drug metabolism may result in residual effects in some participants [31]. Temporary symptom exacerbation during washout could affect task performance and future research should explore optimal washout durations and their impact on diverse ADHD populations to refine study designs. Nevertheless, the waning of training effects without sustained practice [17] highlights the importance of maintenance programs to ensure long-term benefits.

Future research should incorporate no-training control groups and longer training durations (e.g., 8–12 weeks) and follow-ups at 6–12 months to clarify WMT’s specific effects and sustainability [23]. Developing hybrid training programs that target both verbal and visuospatial WM, potentially integrating gamified elements to enhance engagement [51], could improve transfer to daily functioning. Additionally, neuroimaging studies examining prefrontal and parietal activation pre- and post-WMT could elucidate the neural mechanisms underlying training gains [14]. Finally, standardizing Corsi backward protocols across age groups, as suggested by Kessels et al. [6], would enhance its clinical utility in ADHD assessments.

## 5. Conclusions

In conclusion, our results indicate that, in all groups, adaptive dual N-back training significantly enhances working memory performance in divided attention, with large and reliable benefits in working memory scores. Gains in verbal WM in young adults with ADHD, as evidenced by WAIS-IV WMI improvements, were also observed, but are less uniform, and limited visuospatial gains on the Corsi task suggest that changes in traditional cognitive measures (WAIS-IV, Corsi) should be interpreted with more caution. These findings underscore the task-specific nature of WMT and the need for tailored interventions to address ADHD’s heterogeneous cognitive profile. By integrating multimodal training and sustained practice, future interventions can maximize cognitive and functional outcomes for individuals with ADHD.

## Figures and Tables

**Table 1:** Sample Characteristics by Group. EHI = Edinburgh Handedness Inventory; ASRS = Adult ADHD Self-Report Scale; CAARS = Conners’ Adult ADHD Rating Scales. Values represent means ± standard errors.

Group	N	Male	Age	EHI	ASRS	CAARS	Adaptive
		(%)	(M ± SEM)	(M ± SEM)	(M ± SEM)	(M ± SEM)	(%)
CTL	45	51.1	22.58 ± 0.46	82.22 ± 5.51	46.89 ± 1.52	47.31 ± 1.20	46.7
ADHD	33	75.8	22.41 ± 0.67	68.85 ± 9.57	58.36 ± 1.84	57.27 ± 1.42	51.5
MADHD	42	54.8	23.20 ± 0.53	72.38 ± 7.73	65.76 ± 1.87	63.46 ± 1.76	47.6

**Table 2:** ADHD Subtype Distribution by Group (with Percentages). Subtypes are based on DSM-IV criteria: Combined (C), Hyperactive-Impulsive (HI), and Inattentive (I). Unassigned cases refer to participants without a recorded subtype.

Group	Combined (C)	Hyperactive-Impulsive (HI)	Inattentive (I)	Unassigned	Total
ADHD	16 (48.5%)	1 (3.0%)	12 (36.4%)	4 (12.1%)	33
MADHD	28 (66.7%)	0 (0.0%)	10 (23.8%)	4 (9.5%)	42
Total	44 (58.7%)	1 (1.3%)	22 (29.3%)	8 (10.7%)	75

**Table 3:** Summary of behavioral performance scores (M ± SEM) before and after working memory training, reported by Group (Control, ADHD, Medicated ADHD [MADHD]) and Training condition (Fixed-D1B vs. Adaptive). Measures include dual N-back level, WAIS-IV Working Memory subtests, and the Corsi Block-Tapping Task. Statistically significant improvements over baseline (from pairwise two sample *t*-test) are marked with asterisks (* p<0.05; ** p<0.01; *** p<0.001).

Task	Training		Controls				ADHD				MADHD			
			N	Pre	Post	%Δ	N	Pre	Post	%Δ	N	Pre	Post	%Δ
**Dual N-Back (Level)**														
Fixed-D1B			20	2.2	2.9	136.4	15	2.0	2.5	123.4	17	1.8	2.4	132.2
				±0.1	±0.2	±4.3 ***		±0.1	±0.2	±3.5 ***		±0.1	±0.1	±2.6 ***
Adaptive			19	1.9	4.0	204.6	16	1.8	3.4	187.5	19	1.9	3.4	178.8
				±0.1	±0.3	±13.0 ***		±0.1	±0.3	±13.2 ***		±0.1	±0.3	±9.1 ***
**WAIS-IV Working Memory Index**														
*Digit Span Forward*														
Fixed-D1B			20	11.0	11.9	111.8	15	9.9	10.5	107.5	17	9.2	9.7	108.2
				±0.5	±0.5	±6.6		±0.6	±0.6	±3.5 *		±0.5	±0.4	±4.8
Adaptive			19	10.9	10.8	100.0	16	10.1	10.5	103.8	19	10.0	10.9	110.9
				±0.5	±0.5	±3.8		±0.4	±0.6	±3.0		±0.5	±0.5	±4.7 *
*Digit Span Backward*														
Fixed-D1B			20	10.2	10.9	108.5	15	8.7	9.3	107.0	17	8.2	8.6	104.3
				±0.5	±0.5	±3.3 *		±0.6	±0.6	±4.3		±0.3	±0.3	±2.6
Adaptive			19	8.8	10.4	119.4	16	8.6	9.3	109.2	19	9.1	9.4	104.1
				±0.4	±0.5	±4.2 ***		±0.4	±0.5	±4.9		±0.4	±0.5	±3.6
*Digit Span Sequencing*														
Fixed-D1B			20	11.6	12.1	107.5	15	10.7	10.6	100.4	17	8.2	9.1	112.8
				±0.5	±0.4	±4.4		±0.7	±0.7	±6.4		±0.4	±0.5	±5.4 *
Adaptive			19	10.5	11.5	112.4	16	9.4	10.2	111.4	19	10.2	10.9	109.2
				±0.5	±0.5	±7.0		±0.4	±0.6	±7.6		±0.6	±0.7	±5.7
*Total Working Memory Score*														
Fixed-D1B			20	89.6	102.1	115.8	15	72.9	80.0	113.1	17	63.4	67.6	106.7
				±6.6	±7.3	±5.0 **		±8.4	±8.7	±7.7		±3.0	±3.8	±3.3
Adaptive			19	72.6	94.6	135.4	16	69.5	80.6	119.0	19	74.9	80.8	107.6
				±5.2	±6.7	±9.0 ***		±5.8	±7.2	±7.9 *		±5.2	±7.4	±5.5
**Corsi Block-Tapping Task**														
*Number Correct*														
Fixed-D1B			20	11.0	11.9	108.6	15	10.3	11.3	110.2	17	9.8	10.2	105.0
				±0.3	±0.4	±2.9 **		±0.5	±0.6	±5.2		±0.4	±0.4	±3.7
Adaptive			19	11.0	10.8	99.3	16	10.5	10.9	104.6	19	10.2	11.0	109.1
				±0.4	±0.4	±2.9		±0.4	±0.4	± 3.7		±0.5	±0.5	±3.1 **
*Span Length*														
Fixed-D1B			20	7.0	7.5	107.6	15	6.6	7.2	110.8	17	6.2	6.5	106.9
				±0.2	±0.3	±3.7		±0.3	±0.3	±5.6		±0.3	±0.2	±4.2
Adaptive			19	7.2	6.8	95.3	16	6.6	6.9	105.9	19	6.4	6.9	108.3
				±0.2	±0.2	±2.8		±0.2	±0.2	±3.9		±0.3	±0.3	±3.3 *

## Data Availability

All data have been processed following the data security guidelines of the University of Lausanne for human participants. Privacy is regulated by the new Swiss Federal Act on Federal Data Protection (nFADP) (Status of 1 September 2023).
